# Berberine safeguards sepsis‐triggered acute gastric damage and inhibits pyroptosis in gastric epithelial cells via suppressing the ubiquitination and degradation of Nrf2

**DOI:** 10.1002/kjm2.12900

**Published:** 2024-11-01

**Authors:** Shu‐Rui Xie, Yan‐Jun Liu, Fen‐Qiao Chen, Zhao Pan

**Affiliations:** ^1^ Infectious Diseases Department The Fourth Hospital of Hebei Medical University Shijiazhuang Hebei China; ^2^ Emergency Department Hebei Provincial Hospital of Traditional Chinese Medicine Shijiazhuang Hebei China; ^3^ Internal Medicine‐Neurology Hebei Yiling Hospital Shijiazhuang Hebei China

**Keywords:** acute gastric injury, berberine, inflammation, Nrf2, pyroptosis, sepsis

## Abstract

Berberine (BBR), a widely recognized traditional Chinese medicine, has attracted considerable attention for its promising anti‐inflammatory effects. The activation of nuclear factor erythroid 2‐related factor 2 (Nrf2) effectively safeguards against organ damage stemming from sepsis‐induced oxidative stress and inflammatory responses. This study examined the potential of BBR in alleviating sepsis‐induced acute gastric injury, with a particular focus on elucidating whether its mechanism of action involves the activation of the Nrf2 signaling pathway. Following intraperitoneal injection of BBR, mice were subjected to the cecal ligation and puncture (CLP) method to induce sepsis. In vitro experiments involved pre‐treating the normal gastric epithelial cells (GES‐1) with BBR, followed by treatment with lipopolysaccharide (LPS). Functional assays were then performed to assess cell proliferation and apoptosis. To validate the role of Nrf2 in pyroptosis and inflammation, siRNA targeting Nrf2 (si‐Nrf2) was transfected into LPS‐treated GES‐1 cells. Additionally, mice were administered the Nrf2 inhibitor ML385 to confirm the protective effects of BBR in vivo. BBR displayed a dose‐dependent effect in mitigating gastric tissue damage, suppressing the release of inflammatory cytokines, and reducing the expression of NLRP3, ASC, and GSDMD‐N. In vitro, BBR fostered GES‐1 cell proliferation, hindered apoptosis, and suppressed the levels of TNF‐α, IL‐18, IL‐1β, NLRP3, ASC, and GSDMD‐N. Further analysis revealed that knocking down Nrf2 reversed BBR's inhibitory effect on pyroptosis in LPS‐treated GES‐1 cells. Through binding to Keap1, BBR efficiently prevented the ubiquitination and degradation of Nrf2, ultimately promoting its nuclear translocation. In vivo experiments confirmed that ML385 reversed the protective effect of BBR on pyroptosis and inflammation. Our research reveals that BBR interacts with Keap1 to activate the Keap1/Nrf2 signaling pathway in gastric epithelial cells, thereby suppressing pyroptosis and inflammation in sepsis‐induced acute gastric injury.

## 1 INTRODUCTION

Sepsis, an inflammatory response syndrome triggered by invading pathogens, poses significant risks and devastating consequences.^1^ As sepsis progresses to its severe stage, it frequently results in multiple organ dysfunction and circulatory failure, posing a substantial burden on patients and healthcare systems globally.^2^ Despite advancements in understanding its mechanisms, sepsis remains the leading cause of death in intensive care units. The gastrointestinal tract serves as the initial epicenter of multi‐organ failure and is one of the primary systems to encounter impairment.^3^ Sepsis‐induced gastric dysfunction prominently exhibits as disruptions in gastrointestinal motility, nutrient assimilation, and intestinal immune barrier function. These sequelae ultimately culminate in intestinal mucosal atrophy and enteroborne infections, which in turn exacerbate the dysfunction of other organs.^4^ Sepsis complicated with acute gastric injury is associated with a significantly high mortality rate, which stands as a particularly grave concern.^3^, ^5^

Berberine (BBR), an isoquinoline alkaloid, which exists in *Coptis chinensis* and *Berberis vulgaris*, is a well‐recognized component of traditional Chinese herbal medicines.^6^ This alkaloid possesses a diverse array of pharmacological properties, rendering it a versatile therapeutic candidate for treating various medical conditions, including hypertension, diabetes, atherosclerosis, viral pathogen infections, and carcinomas.^7^, ^8^ Recent cellular and animal model studies have delved deeply into the protective effects and mechanisms of BBR in sepsis, highlighting its ability to function as a blocking agent in lipopolysaccharide (LPS)‐provoked sepsis and alleviate sepsis‐linked organ compromise by downregulating inflammatory responses.^9^, ^10^ Nonetheless, the comprehensive understanding of BBR's impact on sepsis‐generated acute gastric injury and its underlying mechanisms remains incomplete.

Pyroptosis, a form of programmed cell death characterized by lytic and inflammatory mechanisms, is typically initiated by inflammasomes and carried out through the activation of gasdermin proteins.^11^ This process is marked by distinctive features such as cell swelling, membrane rupture, and the subsequent release of intracellular contents. The excessive pyroptosis can induce uncontrolled inflammatory responses and participate in the occurrence of inflammatory diseases.^12^ The nod‐like receptor protein 3 (NLRP3) inflammasome, belonging to the NLR family and featuring the pyrin domain, holds a significant position among the most thoroughly investigated inflammasomes.^13^ Once activated, NLRP3 attracts pro‐caspase‐1, resulting in its autocatalytic cleavage and subsequent activation. The activated caspase‐1 catalyzes the maturation of interleukin‐1 beta (IL‐1β) and IL‐18, while also triggering the liberation of Gasdermin D's N‐terminal domain, known as GSDMD‐N.^14^ This liberated domain translocates to the cell membrane, forming pores that facilitate the release of cellular contents, including the aforementioned inflammatory cytokines.^15^ Ultimately, this sequence of events culminates in pyroptosis, a crucial mechanism that underpins excessive inflammatory responses during the initial stages of sepsis.

Nrf2 nuclear translocation is a pivotal step in initiating and amplifying the activation of the Nrf2 signaling pathway.^16^ Upon translocation from the cytoplasm to the nucleus, Nrf2 engages with antioxidant response elements (AREs) within the nucleus, thereby triggering the transcription of vital antioxidant genes such as superoxide dismutase (SOD), glutathione peroxidase (GPx), heme oxygenase‐1 (HO‐1), and numerous others. This orchestrated process significantly bolsters the cell's antioxidant defenses, empowering it to better withstand oxidative stress.^17^ Kelch‐like ECH‐associated protein 1 (Keap1) exerts a negative regulatory influence on Nrf2 activation, specifically impeding the nuclear translocation of Nrf2 under unstressed conditions. Moreover, the Nrf2/Keap1 system acts as a critical modulator in the process of sepsis. Nrf2 was a therapeutic effect of BBR in many diseases, including Alzheimer's disease, nephropathy, and hepatoma.^18^, ^19^, ^20^ However, whether the BBR affects Nrf2 signaling in sepsis‐triggered acute gastric damage is not yet known.

The present research is to clarify the protective action of BBR in sepsis‐triggered acute gastric injury, while also delving into the potential mechanisms that underlie BBR's modulation of pyroptosis and inflammatory reactions within gastric tissues.

## 2 MATERIALS AND METHODS

### 2.1 Animal model

Eight‐week‐old male C57BL/6 mice were housed in pathogen‐free environment, granted unrestricted feeding, and maintained on a 12‐h light/dark cycle. All experimental protocols involving the mice were authorized by the Institutional Animal Care and Use Committee (No. HBMU‐IACUC‐2022‐11‐19). After a week of acclimatization, the mice were randomly distributed into five groups, each comprising six animals: Sham, CLP, and three BBR‐treated groups, receiving dosages of (20, 50, and 100) mg/kg, respectively. To establish the sepsis model, mice were subjected to cecal ligation and puncture (CLP) surgery. Prior to the procedure, mice were anesthetized via intraperitoneal injection of 3% pentobarbital sodium (50 mg/kg).^21^ A midline incision was performed to expose the abdominal cavity, and the cecum was delicately separated from neighboring tissues. The cecum was then ligated in its middle section and punctured five times with a sterile needle, ensuring the expulsion of a small amount of fecal matter from the large intestine. Following this, the cecum was repositioned, and the abdominal cavity was sutured closed. In contrast, mice in the sham group underwent a similar laparotomy but without the CLP procedures. Regarding BBR (#14050, Sigma, USA) treatment, it was dissolved in saline solution and administered intraperitoneally to mice in the low (20 mg/kg), medium (50 mg/kg), and high (100 mg/kg) dosage groups, 48 h prior to the CLP surgery. Additionally, the Nrf2 inhibitor ML385 (30 mg/kg, #S8790, Selleck, USA) was administered intraperitoneally to the mice 3 h before the CLP surgery. All mice, regardless of the group, were euthanized 24 h following the CLP or sham surgical procedure. Serum aspartate aminotransferase (AST) and alanine aminotransferase (ALT) levels in mice were evaluated using an automated biochemical analyzer (Roche Cobas 8000 System, Mannheim, Germany).

### 2.2 Hematoxylin–eosin (H&E) staining

The excised mouse gastric tissues were fixed in formalin solution for 48 h. After fixation, the tissues underwent a dehydration process involving graduated concentrations of alcohol, culminating in paraffin embedding. The embedded tissues were then precisely sliced into 4‐μm‐thick sections. To prepare the tissue sections for microscopic examination, they were initially dewaxed with xylene and subsequently rehydrated through a graded ethanol series. Following this, hematoxylin was applied to visualize the nuclei, and eosin served as a counterstain for the cytoplasm. Ultimately, the stained sections were scrutinized on an optical microscope (Nikon, Tokyo, Japan) to evaluate any pathological alterations in the gastric tissues.

### 2.3 Immunohistochemistry staining

To prepare the tissue sections for immunohistochemical analysis, the initial steps involved dewaxing and rehydrating the sections through a series of graded solutions. Subsequently, a 10‐min incubation with 3% H_2_O_2_ at room temperature was performed. Following this, the sections were incubated overnight with an anti‐GSDMD‐N antibody (#AWA10007, 1:200, Abiowell Biotechnology, Changsha, China). Subsequently, the samples were incubated with an HRP‐labeled IgG secondary antibody for 10 min at 37°C. The sections were then counterstained with hematoxylin to enhance contrast and reveal structural details. Images were captured using a Nikon light microscope for a comprehensive evaluation of GSDMD‐N expression.

### 2.4 Cell viability assay

The CCK‐8 assay was performed to measure the viability of GES‐1 cells. Initially, the cells were seeded in 96‐well plates and cultured overnight to ensure proper adherence and growth. Following this, the cells were stimulated with indicated doses of BBR (2, 5, and 10 μM) for 6 h. Subsequently, 1 μg/mL of LPS was added to cells and maintained for 24 h. After the LPS treatment, 10 μL of the CCK‐8 reagent (#CA1210, Solarbio, Beijing, China) was added to initiate the viability assay. Cells were then kept at 37°C for 2 h in a dark environment to allow for the reaction to occur. Finally, a microplate reader was utilized to assess the absorbance (OD) at 450 nm, which provided a quantitative assessment of the cell viability.

### 2.5 5‐Ethynyl‐2′‐deoxyuridine assay

To assess cell proliferation, both transfected and untransfected GES‐1 cells were seeded into 96‐well plates, alongside varying concentrations of LPS and BBR. Following 24 h of culture, the medium was discarded and refreshed with 10 μM 5‐ethynyl‐2′‐deoxyuridine assay (EdU) solution (Beyotime, Shanghai, China) for a 2‐h incubation period. Cells were then washed with PBS and fixed with 4% paraformaldehyde for 30 min to immobilize them. Following this, 0.3% Triton X‐100 was used to permeabilize the cells for 10 min, allowing for the penetration of staining reagents. Next, DAPI (Beyotime) was added to stain the cell nuclei at 37°C for 10 min. Finally, a fluorescence microscope was utilized to quantify the percentage of EdU‐positive cells, providing a quantitative measure of cell proliferation.

### 2.6 Apoptosis assay

To detect cell apoptosis, an Annexin V‐FITC/PI Apoptosis Detection Kit from Abcam (USA) was employed. Initially, the cells were washed with cold PBS to eliminate any residual media or debris. The cells were resuspended in annexin‐binding buffer. Then, 10 μL of Annexin V‐FITC and 5 μL of PI were added to ensure adequate labeling. The cells were incubated in the dark for 10 min to allow for the binding of the Annexin V‐FITC and PI. Finally, a flow cytometer (BD Biosciences, USA) was employed to evaluate and quantify the apoptotic cells.

### 2.7 Enzyme‐linked immunosorbent assay

To extract the tissue homogenate from mouse gastric tissues, a lysis buffer was used. This homogenate was then centrifuged to separate components. The resulting cell supernatants were collected and further centrifuged to eliminate any cell debris. To detect the presence of TNF‐α, IL‐1β, and IL‐8 in the mouse gastric tissues and GES‐1 cells, an ELISA kit from R&D Systems Inc. (Minneapolis, USA) was employed, following the manufacturer's instructions. A microplate reader from Bio‐TEK (USA) was utilized to measure the absorbance at 450 nm, thereby quantifying the levels of the inflammatory cytokines.

### 2.8 Western blot analysis

The mouse gastric tissues were homogenized, and GES‐1 cells were gathered. Both tissues and cells underwent lysis in RIPA buffer on ice for 15 min to extract the total protein content. The lysates were then centrifuged to isolate the total protein. Utilizing the BCA protein quantification kit from Beyotime (Shanghai, China), the protein concentration was determined. For electrophoresis, 30 μg of protein was loaded onto SDS–PAGE gels and then transferred onto PVDF membranes. Following blocking, an overnight incubation at 4°C with primary antibodies specific to NLRP3 (#ab263899, 1:1000, Abcam), GSDMD‐N (#AWA10007, 1:1000, Abiowell Biotechnology), ASC (#ab309497, 1:1000, Abcam), and GAPDH (#ab8245, 1:3000, Abcam) was performed. After three washes with TBST, the membranes were incubated with secondary antibodies (#ab288151, 1:3000, Abcam) for 1 h. Protein bands were visualized using enhanced chemiluminescence reagents from Bio‐Rad Laboratories, Inc. Finally, the band densities were analyzed with the Bio‐Rad Image System to quantitatively assess and compare the protein expression levels of interest.

### 2.9 Quantitative reverse transcription PCR

To extract total RNA from GES‐1 cells, TRIzol reagent was utilized. This RNA was then converted into cDNA using the cDNA Synthesis kit from Takara (Otsu, Shiga, Japan). For quantitative real‐time PCR (qRT‐PCR) detection, SYBR Green qPCR Master Mix from MedChemExpress (NJ, USA) was chosen. The relative expression level of the target genes was calculated using the 2^−ΔΔCT^ method. GAPDH was served as a reference gene for normalization.

### 2.10 Molecular docking

To determine the initial structure of the Nrf2/BBR complex for further analysis, we utilized Discovery Studio 3.1 software. Firstly, we obtained the crystal structure of the Nrf2/Keap1 complex from the Protein Data Bank database (PDB ID: 1X2R). To establish the minimum energy conformations for molecular dynamics (MD) simulations, we employed the default parameters PyMoL (version 1.7.6) provided by the software. The molecular structures of Nrf2–Keap1 and BBR's were generated for MD analysis using AutoDockTools (version 1.5.6).

### 2.11 Detection of caspase‐1 activity

After being treated with LPS and BBR, GES‐1 cells were gathered and labeled with the FAM‐YVAD‐FMK kit (#653, AmyJet, Wuhan, China) for 1 h. Subsequently, the cells were washed, and examined under a fluorescence microscope. The presence of brightly green‐fluorescing cells served as an indicator of increased caspase‐1 activity.

### 2.12 Co‐immunoprecipitation and ubiquitination assays

Cells were incubated in 300 μL of lysis buffer on ice for 5 min to enable cell lysis and subsequent protein extraction. The cell lysates were then combined with a primary antibody specific to the target protein and incubated overnight at 4°C to facilitate binding. Subsequently, 50 μL of protein G agarose beads were introduced to the mixture to capture the formed antigen–antibody complexes. After thorough washing with lysis buffer three times, the immunoprecipitates were separated by centrifugation at 10,000 rpm for 1 min. To denature the proteins and release them from the beads, the samples were heated in an appropriate buffer. Following this, a western blot assay was conducted to detect the presence of the target protein. Specifically, an anti‐ubiquitin antibody was employed to identify ubiquitinated Nrf2, a marker of its potential degradation.

### 2.13 Cycloheximide and MG132 assays

Cycloheximide (CHX) or MG132 were introduced to the cell culture medium at a final concentration of 10 μM for CHX and 10 μM for MG132. Afterward, cell lysates were collected at designated time points: 0, 12, and 24 h, following the application of CHX or MG132.

### 2.14 Statistical analysis

Statistical analyses were carried out utilizing SPSS 21.0 software (IBM Corp., Armonk, NY, USA). All data are presented as mean values with their corresponding standard deviations (SD). To compare differences among multiple groups, a one‐way ANOVA was employed, and Tukey's post hoc tests were subsequently performed. The survival outcomes were analyzed using Kaplan–Meier curves and the log‐rank test. A *p*‐value less than 0.05 was deemed statistically significant.

## 3 RESULTS

### 3.1 BBR ameliorates sepsis‐related mice acute gastric injury

**FIGURE 1:**
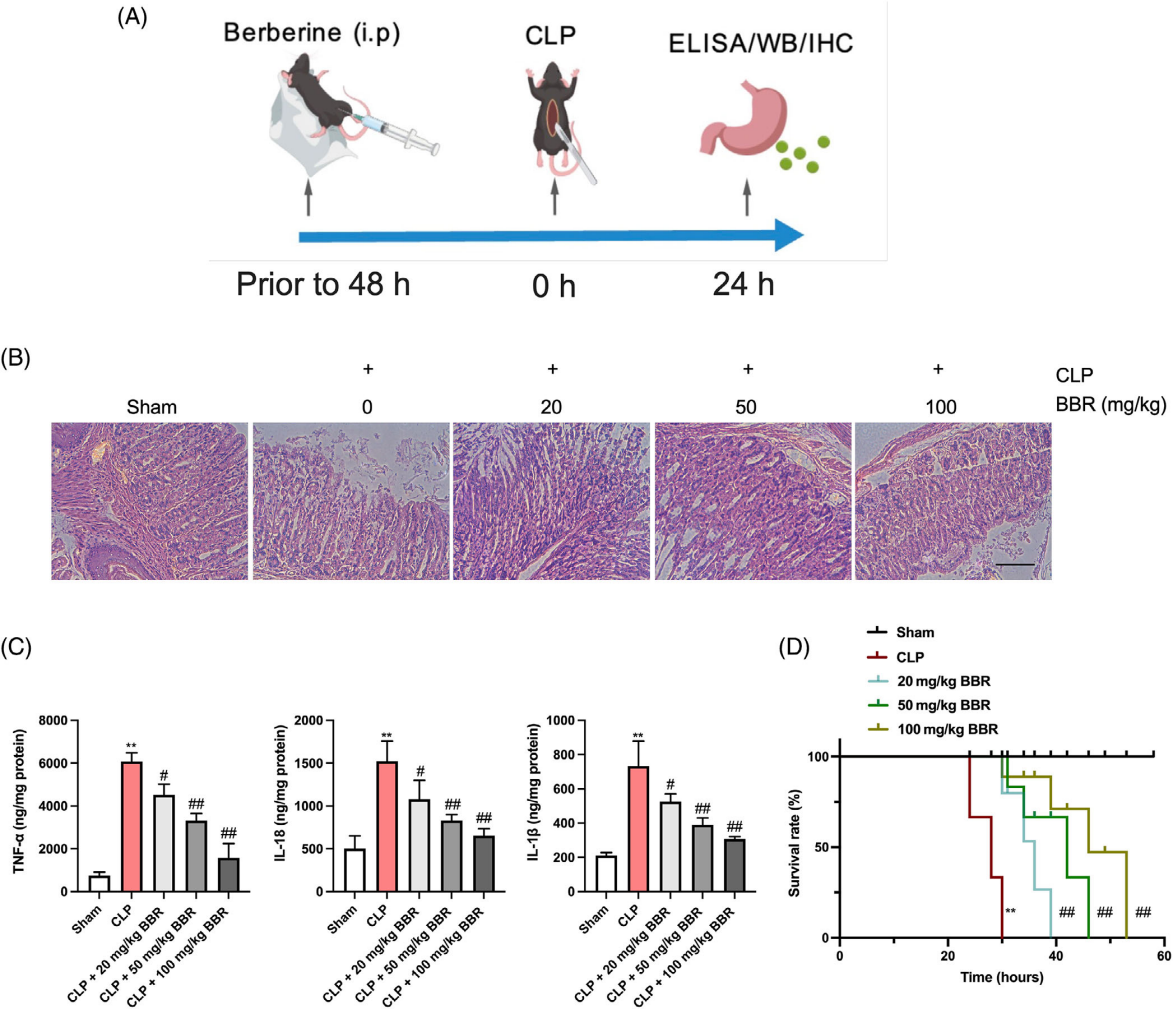
BBR ameliorates sepsis‐related mouse acute gastric injury. Mice were injected intraperitoneally with BBR at low (20 mg/kg), medium (50 mg/kg), and high (100 mg/kg) doses before CLP surgery. *N* = 6. (A) The schematic diagram of experimental process progress. (B) H&E staining was performed to examine histopathological damage in gastric tissues (200×). Scale bar: 100 μm. (C) ELISA detection of TNF‐α, IL‐18, and IL‐1β in gastric tissue. (D) Survival rate of mice in each group. Log‐rank test was employed for the comparison among groups. *N* = 13. **p < 0.01 versus Sham group; ^#^ *p* < 0.05, ^##^ *p* < 0.01 versus CLP group.

To assess the potential therapeutic effects of BBR in sepsis, we administered various doses of BBR to mice prior to establishing a CLP‐induced sepsis model. The experimental protocol is schematically depicted in Figure 1A. Mice destined for sepsis induction were pretreated with BBR at low (20 mg/kg), medium (50 mg/kg), and high (100 mg/kg) doses before undergoing CLP surgery. As illustrated in Figure 1B, the sham group and sham + BBR (100 mg/kg) group exhibited a healthy gastric mucosa and submucosa structure, in contrast to the model group, which exhibited denatured and necrotic superficial gastric mucosal epithelial cells, along with loosely arranged deep cells and interstitial edema. However, with increasing doses of BBR supplementation, these histopathological injuries were mitigated compared to the model group. To assess the potential toxic and adverse effects of BBR on normal mice, we administered a dose of 100 mg/kg of BBR to the mice through intraperitoneal injection. Following a 48‐h period, serum ALT and AST levels, as well as the major organs (heart, liver, kidney, lung, and gastric tissue) were evaluated. Serum ALT and AST levels were measured to determine the toxicity of BBR. No significant difference was observed on serum ALT and AST levels between control group and BBR (100 mg/kg) group (Figure S1). Furthermore, the H&E staining of heart, liver, kidney, lung, and gastric tissue from the 100 mg/kg BBR group exhibited no pathological alterations (Figure S1). The development of sepsis‐related acute gastric injury is often accompanied by inflammatory responses.^22^ We therefore investigated the impact of BBR on inflammatory cytokines in the gastric tissues of the model mice. ELISA analysis revealed that CLP induced an upregulation of TNF‐α, IL‐18, and IL‐1β levels in the gastric tissues, while these increases were attenuated after treatment with the indicated doses of BBR (Figure 1C). Additionally, we detected the gastric injury at different time points, and the results showed that the gastric tissue structure damage and TNF‐α, IL‐18, and IL‐1β release were significant at 12 h, and more significant at 24 h after modeling (Figure S2A,B). Moreover, the survival rate among mice in the BBR treatment groups was notably superior to that of the model group (Figure 1D). Our results indicate that BBR exhibits promising therapeutic potential in mitigating sepsis‐induced acute gastric injury and inflammation.

### 3.2 BBR inhibits NLRP3‐mediated pyroptosis in sepsis‐related acute gastric injury

**FIGURE 2:**
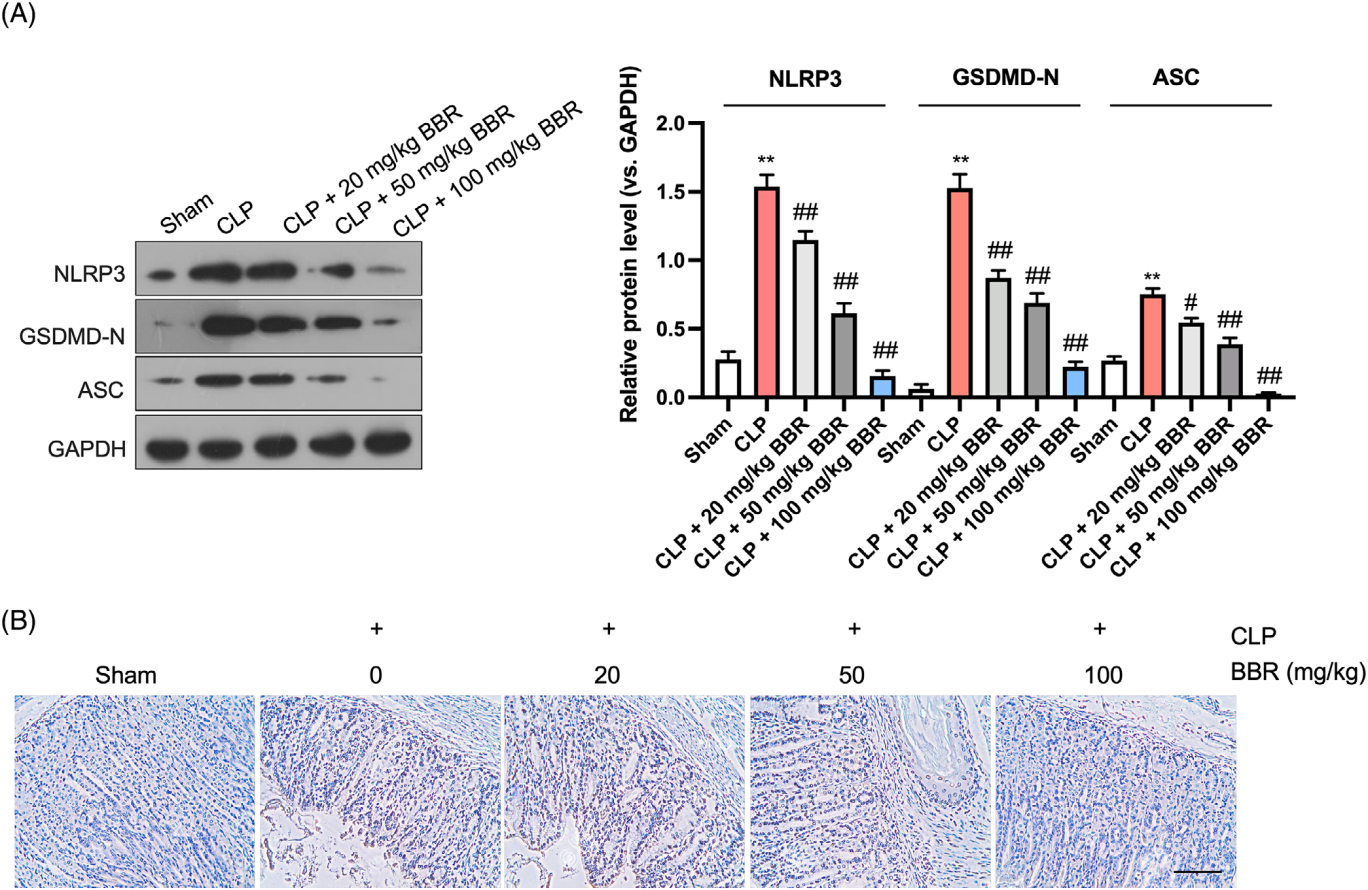
BBR inhibits NLRP3‐mediated pyroptosis in sepsis‐related mouse acute gastric injury. (A) Western blotting detection of NLRP3, ASC, and GSDMD‐N in gastric tissues. (B) Immunohistochemical staining of GSDMD‐N protein in gastric tissues (200×). Scale bar: 100 μm. *N* = 6.

To investigate the influence of BBR on pyroptosis during sepsis‐induced acute gastric injury, we evaluated markers linked to NLRP3 activation and pyroptosis. Western blot analysis demonstrated a substantial elevation in the expression of NLRP3, ASC, and GSDMD‐N in gastric tissues, which was subsequently mitigated in a dose‐dependent fashion following BBR treatment (Figure 2A). Consistently, immunohistochemical staining results also supported these observations, revealing that the CLP‐induced upregulation of GSDMD‐N protein was reversed by BBR administration at varying concentrations (Figure 2B). Collectively, our results underscore the suppressive effect of BBR on pyroptosis in sepsis‐induced acute gastric injury.

### 3.3 BBR suppresses pyroptosis and inflammation in LPS‐induced gastric epithelial cells

**FIGURE 3:**
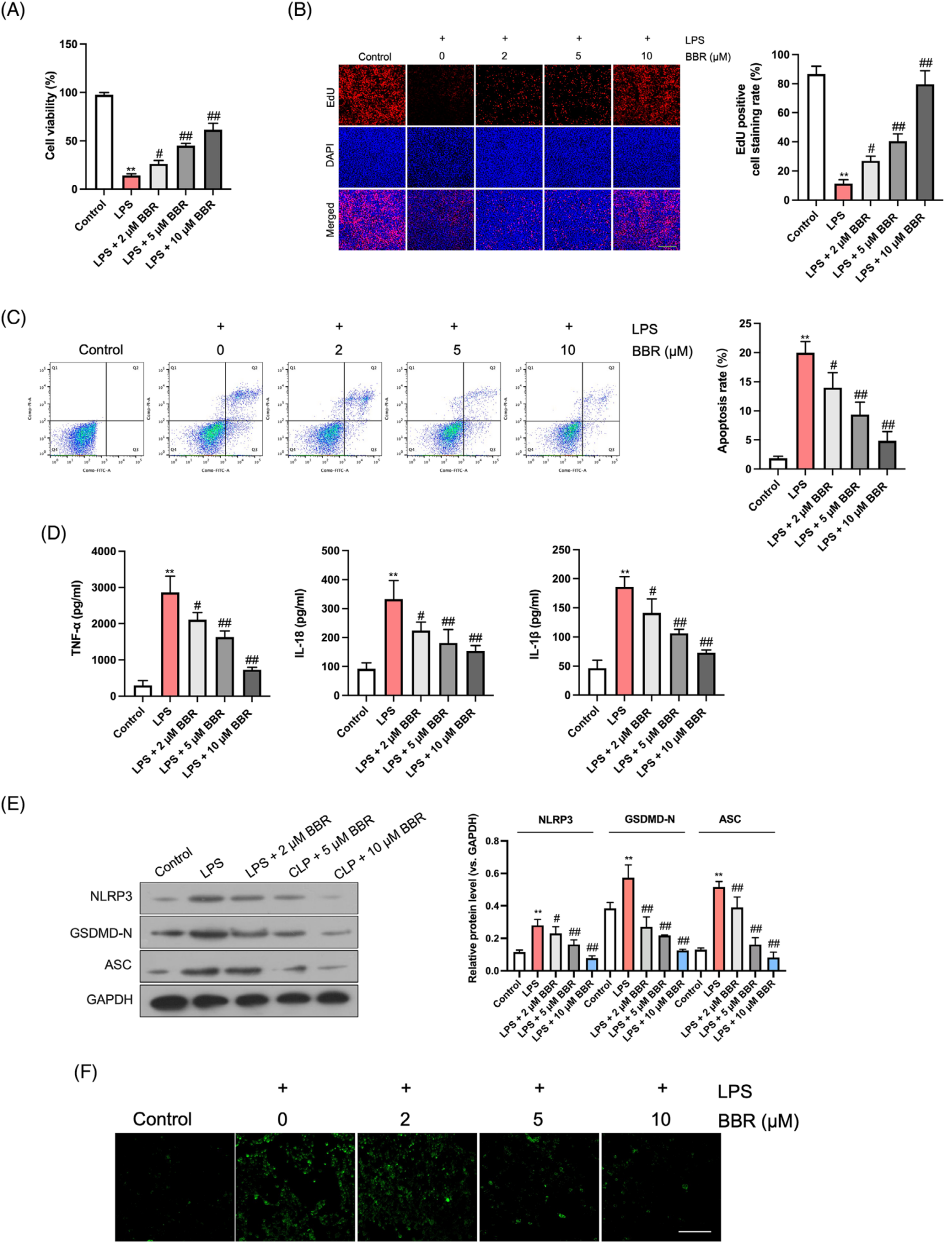
BBR suppresses pyroptosis and inflammation in LPS‐induced gastric epithelial cells. GES‐1 cells were pre‐treated with BBR (2, 5, and 10 μM) for 6 h followed by LPS (1 μg/mL) treatment for 24 h. (A) CCK‐8, (B) Edu, and (C) flow cytometry assays were performed to measure cell proliferation and apoptosis. Scale bar: 100 μm. (D) ELISA was used to measure TNF‐α, IL‐18, and IL‐1β levels in cell supernatants. (E) Western blotting assay for the protein levels of NLRP3, ASC, and GSDMD‐N. (F) The fluorescence intensity of caspase‐1 was measured. *N* = 3. Scale bar: 100 μm. **p < 0.01 versus control group; ^#^ *p* < 0.05, ^##^ *p* < 0.01 versus LPS group.

To replicate an in vitro sepsis‐like environment, the GES‐1 cells were pretreated with BBR prior to exposure to LPS. CCK‐8 and EdU tests revealed a considerable impediment in the proliferation and growth of GES‐1 cells induced by LPS, which was alleviated in a dose‐dependent fashion following BBR treatment (Figure 3A,B). Flow cytometry analysis further confirmed that LPS augmented cell apoptosis, an effect that was attenuated by BBR in a dose‐dependent manner (Figure 3C). Additionally, LPS stimulation significantly elevated the concentrations of inflammatory factors TNF‐α, IL‐18, and IL‐1β in cell supernatants, yet this upregulation was mitigated by increasing doses of BBR (Figure 3D). In line with these findings, western blot analysis demonstrated that LPS upregulated the degrees of the pyroptosis markers NLRP3, ASC, and GSDMD‐N. However, BBR supplementation at the indicated doses reversed these effects (Figure 3E). Furthermore, immunofluorescent staining of caspase‐1 in GES‐1 cells revealed a substantial increase in fluorescence intensity in the LPS group compared to the control group. However, BBR treatment reduced the fluorescence intensity of caspase‐1 in a dose‐dependent manner (Figure 3F). Collectively, these comprehensive data suggest that BBR effectively suppresses pyroptosis and inflammation in gastric epithelial cells induced by LPS.

### 3.4 BBR represses pyroptosis in LPS‐induced GES‐1 cells via activation of Keap1/Nrf2 signaling pathway

**FIGURE 4:**
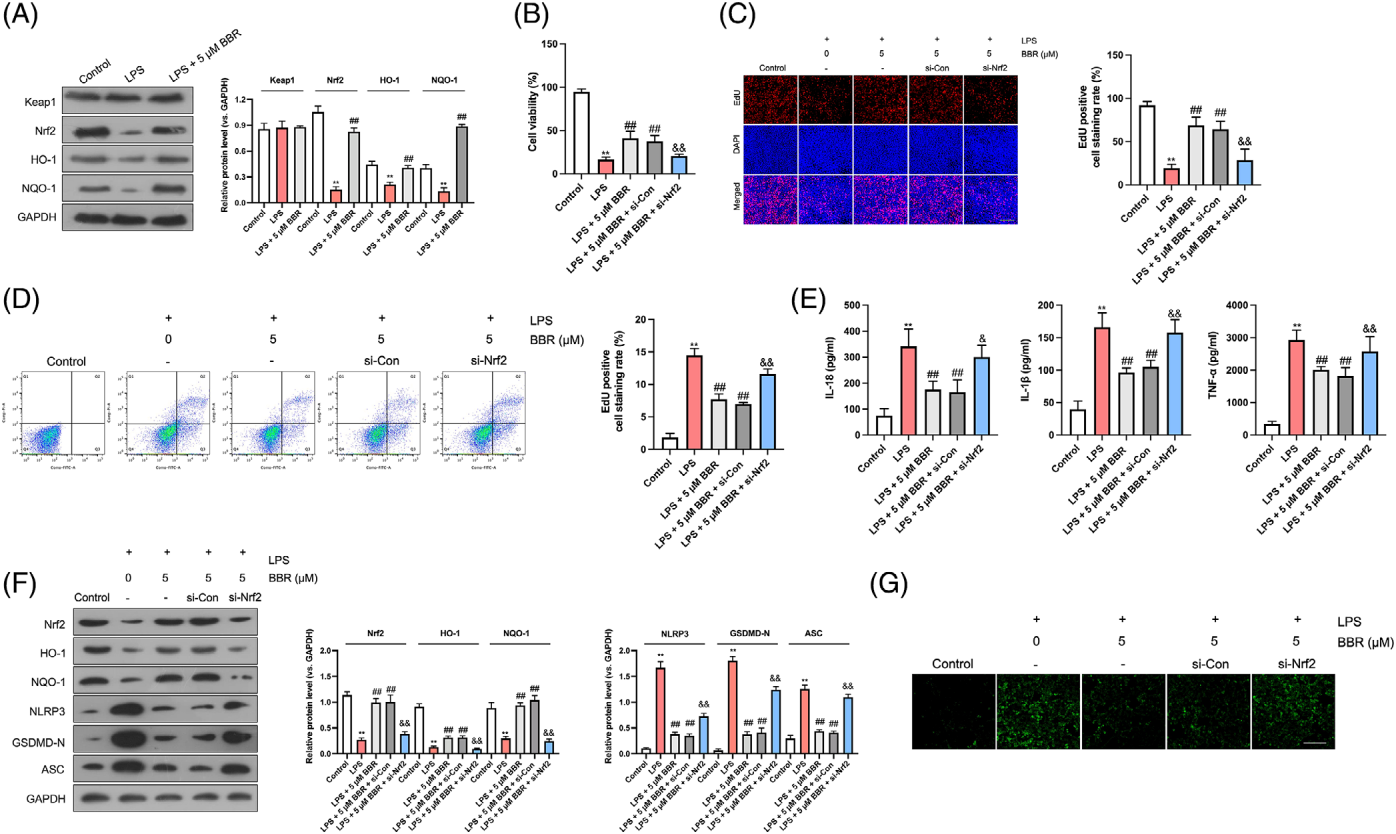
BBR represses pyroptosis in LPS‐induced gastric epithelial cells via activation of Keap1/Nrf2 signaling pathway. GES‐1 cells were pre‐treated with BBR (5 μM) for 6 h followed by LPS (1 μg/mL) treatment for 24 h. (A) Western blotting detection of Keap1, Nrf2, HO‐1, and NOQ‐1. (B–D) The si‐Nrf2 or si‐Con was transfected into GES‐1 cells, following BBR and LPS stimulation. CCK‐8, Edu, and flow cytometry assays were employed to detect cell proliferation and apoptosis. Scale bar: 100 μm. (E) ELISA detection of TNF‐α, IL‐18, and IL‐1β in cell supernatants. (F) Western blotting for NLRP3, ASC, and GSDMD‐N. (G) The fluorescence intensity of caspase‐1 was measured. *N* = 3. Scale bar: 100 μm. **p < 0.01 versus control group; ^##^ *p* < 0.01 versus LPS group; ^&^ *p* < 0.05, ^&&^ *p* < 0.01 versus LPS + 5 μM BBR group.

To further elucidate the mechanistic underpinnings of how BBR regulates pyroptosis in vitro, GES‐1 cells were pretreated with BBR prior to LPS exposure. As illustrated in Figure 4A, LPS treatment alone did not significantly alter Keap1 protein levels but decreased the expressions of Nrf2, HO‐1, and NOQ1. However, upon BBR supplementation, a significant enhancement in Nrf2, HO‐1, and NOQ1 expressions was observed, with minimal changes in Keap1 protein levels. To investigate whether Nrf2 signaling mediates BBR's protective effects on GES‐1 cell proliferation, Nrf2 was knocked down in LPS‐treated cells (data not shown). Our findings revealed that compared to the LPS + BBR group, the LPS + BBR + si‐Nrf2 group exhibited reduced cell proliferation (Figure 4B,C), augmented cell apoptosis (Figure 4D), and heightened levels of inflammatory cytokines TNF‐α, IL‐18, and IL‐1β (Figure 4E). Furthermore, Nrf2 knockdown reversed the suppressive effects of BBR on the protein expressions of NLRP3, ASC, GSDMD‐N, and caspase‐1 level in LPS‐treated GES‐1 cells (Figure 4F,G). In summary, these results suggest that BBR's suppressive actions on pyroptosis and inflammation in LPS‐induced gastric epithelial cells are mediated through modulation of the Keap1/Nrf2 signaling cascade.

### 3.5 BBR inhibits the ubiquitination of Nrf2 through binding to Keap1

**FIGURE 5:**
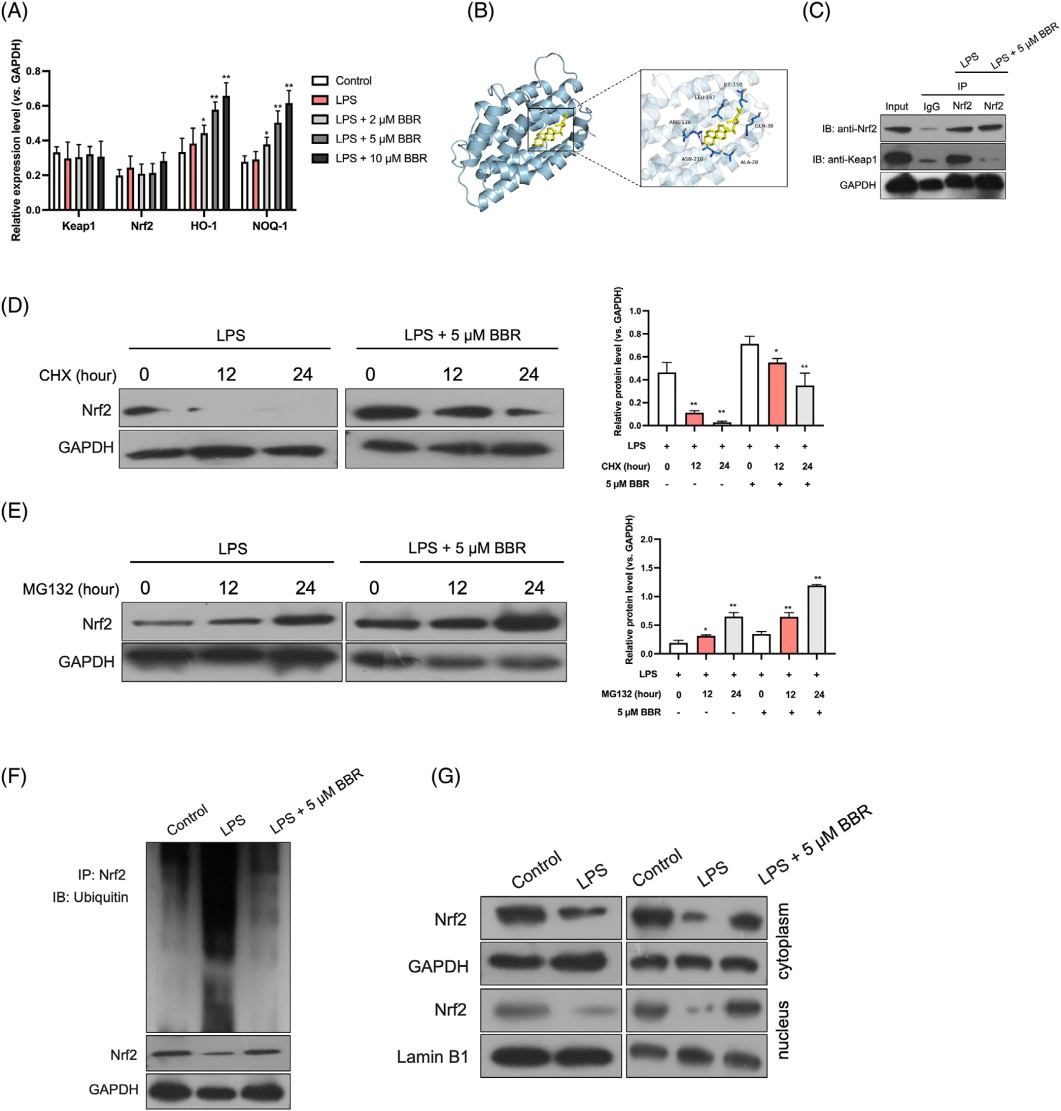
BBR inhibits the ubiquitination of Nrf2 through binding to Keap1. GES‐1 cells were pre‐treated with BBR (5 μM) for 6 h followed by LPS (1 μg/mL) treatment for 24 h. (A) qRT‐PCR analysis for mRNA expressions of Keap1, Nrf2, HO‐1, and NOQ‐1. (B) Autodock analysis for the binding of BBR and Keap1. (C) Co‐IP assay was conducted to confirm the interaction of BBR and Keap1. (D) CHX (10 μM) was added to cells and incubated for a specified period. Nrf2 protein level was measured. (E) MG132 (10 μM) was used to incubate with cells. Nrf2 protein level was measured. (F) The ubiquitination level of Nrf2 was validated. (G) The nuclear and cytoplasm of GES‐1 cells was separated. Nrf2 protein level in nuclear and cytoplasm was measured. *N* = 3. *p < 0.05, **p < 0.01 versus LPS group.

The qRT‐PCR analysis revealed that BBR supplementation did not affect the mRNA expressions of Keap1 and Nrf2 but significantly upregulated the mRNA levels of their downstream targets, HO‐1 and NOQ1 (Figure 5A). Further autodock analysis indicated that compared with Nrf2 and HO‐1, the binding affinity of BBR to Keap1 is higher. The binding affinity was found to be −8.4 kcal/mol, and BBR showed favorable embedding into the Keap1 structural domain, with local interaction images showing hydrogen bonds between BBR and Keap1 was formed (Figure 5B). To experimentally validate this interaction, a Co‐IP assay demonstrated that BBR disrupt the Keap1–Nrf2 complex (Figure 5C). To assess the effect of BBR on Nrf2 protein stability, we employed CHX and MG132 to inhibit protein synthesis and degradation, respectively. CHX treatment led to a reduction in Nrf2 protein levels, which was mitigated by BBR treatment (Figure 5D). Conversely, MG132 blocked Nrf2 degradation, resulting in elevated Nrf2 levels, which were further augmented by BBR (Figure 5E). Subsequent analysis revealed that LPS treatment enhanced the ubiquitination of Nrf2, an effect that was attenuated by BBR (Figure 5F). To evaluate Nrf2's nuclear translocation, we separated cytoplasmic and nuclear fractions and quantified Nrf2 level. Our results showed that BBR treatment significantly increased the nuclear accumulation of Nrf2 (Figure 5G). Collectively, these findings indicate that BBR binds to Keap1, inhibits Nrf2 ubiquitination, and thereby activates Nrf2 signaling.

### 3.6 Nrf2 inhibitor reverses the effect of BBR on pyroptosis and inflammation in sepsis‐related acute gastric injury

**FIGURE 6:**
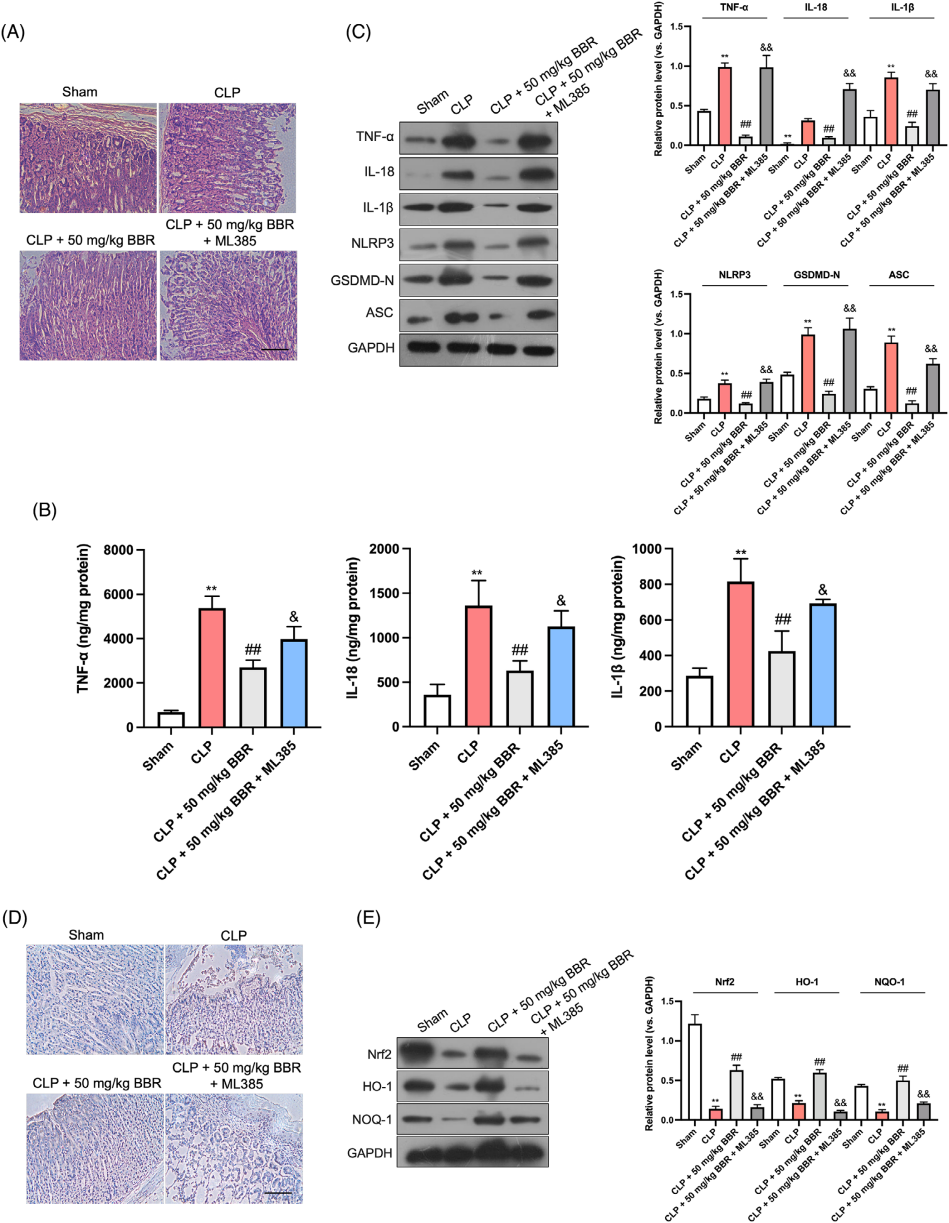
Nrf2 inhibitor reverses the effect of BBR on pyroptosis and inflammation in sepsis‐related mouse acute gastric injury. (A) H&E staining on gastric tissue sections (200×). Scale bar: 100 μm. (B) ELISA detection of TNF‐α, IL‐18, and IL‐1β in gastric tissues. (C) Western blotting detection of NLRP3, ASC, and GSDMD‐N. (D) Immunohistochemical staining of GSDMD‐N protein in gastric tissues (200×). Scale bar: 100 μm. (E) Western blotting detection of Nrf2, HO‐1, and NOQ‐1. *N* = 6. **p < 0.01 versus Sham group; ^##^ *p* < 0.01 versus CLP group; ^&^ *p* < 0.05 versus CLP + 50 mg/kg BBR group.

**FIGURE 7:**
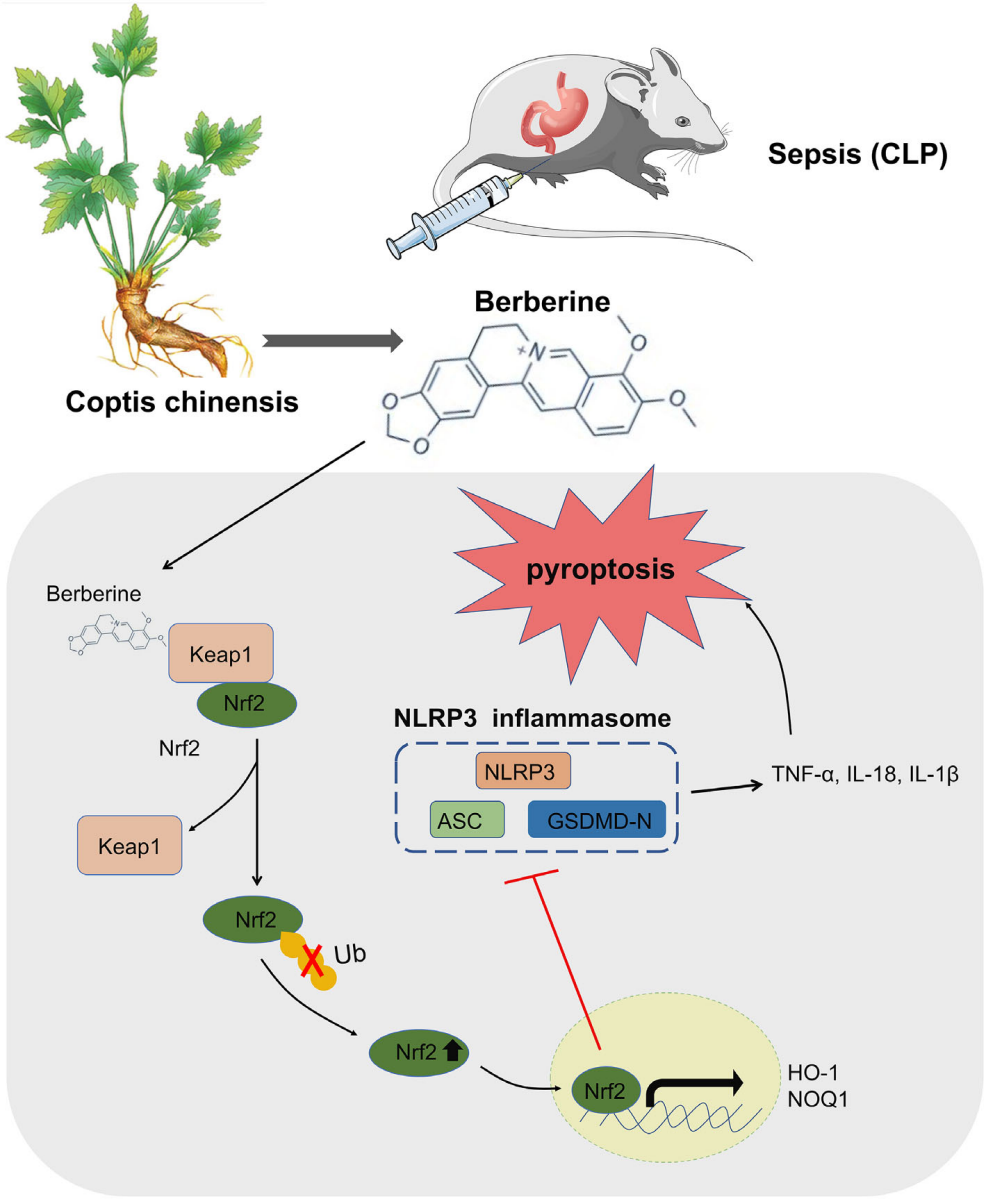
The protective effect and mechanism of berberine for sepsis‐related gastric injury. Berberine binds to Keap1, inhibiting the ubiquitination of Nrf2, thereby activating the Keap1/Nrf2 signaling pathway and subsequently reducing sepsis‐induced gastric injury.

In vivo studies involving mice demonstrated that prior intraperitoneal administration of an Nrf2 inhibitor, ML385 (30 mg/kg), 3 h before CLP surgery mitigated the beneficial effects of subsequent 50 mg/kg BBR treatment. Histopathological analysis using H&E staining revealed that BBR supplementation significantly reduced tissue damage. However, pretreatment with ML385 negated these protective effects of BBR (Figure 6A). Furthermore, ML385 reversed the suppressive effect of BBR on the release of inflammatory cytokines, including TNF‐α, IL‐18, and IL‐1β (Figure 6B). It also abrogated the downregulation of pyroptosis markers, such as NLRP3, ASC, and GSDMD‐N, observed in BBR‐treated mice subjected to CLP (Figure 6C,D). Western blot analysis confirmed that the upregulation of Nrf2, HO‐1, and NOQ‐1 proteins observed in BBR‐treated mice with CLP was negated by ML385 pretreatment (Figure 6E). In summary, these findings underscore the pivotal role of Nrf2 signaling in mediating the suppressive effects of BBR on pyroptosis and inflammation in sepsis‐induced acute gastric injury (Figure 7).

## 4 DISCUSSION

Patients with sepsis are usually accompanied by multiple organ damage, and the stomach is an organ susceptible to sepsis.^4^ In critically patients, gastric injury is a common organ with high incidence, attracts widely attention in clinical.^3^ LPS treatment in mice triggers a heightened inflammatory response, characterized by a swift escalation of serum proinflammatory cytokine levels.^23^ This response is characterized by an earlier onset and a more pronounced expression of cytokines in mice, with the inflammatory process typically lasting a shorter duration compared to humans.^24^ Consequently, the LPS model falls short in fully replicating the complexities of human sepsis. Conversely, the CLP model stands as the prevalent choice for sepsis modeling due to its remarkable compatibility with the human condition.^25^ Following CLP induction, it elicits immunological, hemodynamic, and biochemical alterations that closely mimic those observed in human sepsis, fostering a more protracted inflammatory cascade.^26^ This research adopts the CLP method for inducing mouse septic model, the model group showed significantly histopathological injuries and increased levels of inflammation cytokines TNF‐α, IL‐18, and IL‐1β in gastric tissues, as well as reduced survival rate of mice, indicating the successful preparation of the mouse model of acute gastric injury induced by sepsis.

BBR, an isoquinoline alkaloid found in traditional Chinese herbs like *Coptis chinensis* and *Phellodendron amurense*, has long been utilized clinically for its heat‐clearing, detoxifying, and antibacterial properties.^27^ However, recent research has expanded our understanding of its pharmacological potential, revealing that BBR also possesses benefits such as lipid‐lowering, antioxidant, and anti‐inflammatory characteristic.^28^, ^29^ The protective effect of BBR has been reported on sepsis‐induced organ damage, including intestinal barrier injury, lung injury, acute kidney injury, and liver injury.^30^ In a study undertaken by Wang et al., BBR was shown to function as a negative regulator in sepsis‐induced lung injury, significantly reducing the release of inflammatory cytokines like TNF‐α, IL‐6, and IL‐1β.^31^ Given that IL‐1β, TNF‐α, and IL‐6 are crucial cytokines that play pivotal roles in the pathophysiology of sepsis,^32^ the current investigation demonstrates that BBR, in a dose‐dependent manner, alleviates histopathological damage to gastric tissue, reduces the release of these inflammatory cytokines, and enhances survival rates in septic mice. The obtained results suggest that BBR exerts a beneficial protective effect against gastric injury induced by sepsis.

The pyroptosis depends on the Gasdermin family protein membrane pore formation, and the activation of caspase and GSDMD and release of proinflammatory factors indicate the pyroptosis occurred.^33^ NLRP3 inflammasome including NLRP3, ASC, and caspase‐1, and the dissociation of NLRP3 inflammasome activates caspase‐1, thereby initiating the classical caspase‐1‐dependent pyroptosis pathway.^34^ Activated caspase‐1 dissociates the N‐terminal domain of GSDMD, which destroys the integrity of the cell membrane, and then releases cytokines such as IL‐1β and IL‐18, leading to pyroptosis.^35^ Studies have found that NLRP3/caspase‐1/GSDMD‐mediated pyroptosis plays an important role in the occurrence and development of sepsis, and inhibiting NLRP3 inflammasome‐mediated pyroptosis can reduce acute lung injury in sepsis.^36^ Nevertheless, its role in sepsis‐induced acute gastric injury remains unclear. Our observations revealed an upregulation of the pyroptosis markers NLRP3, ASC, and GSDMD‐N in gastric tissues of mice subjected to CLP and in LPS‐treated GES‐1 cells. Notably, BBR displayed a dose‐dependent inhibition of these markers. Similar to our data, Ding et al. demonstrated that BBR could reduce renal cell pyroptosis in golden hamsters with diabetic nephropathy.^37^ An et al. reported that BBR ameliorated pulmonary inflammation in mice with influenza viral pneumonia by inhibiting NLRP3‐mediated pyroptosis.^38^ This evidence suggests the inhibitory effect of BBR on pyroptosis, which was consistent to our data. The unchecked inflammatory reactions in tissues have the potential to trigger cell apoptosis.^39^ It was next clarified that BBR markedly inhibited apoptosis of GES‐1 cells and promoted proliferation. The above findings indicated that BBR inhibited pyroptosis and inflammation in sepsis‐induced gastric injury.

To validate the regulatory mechanism of BBR's effect on pyroptosis and inflammation, we silenced Nrf2 in LPS‐stimulated GES‐1 cells. Our results showed that BBR significantly upregulated the protein expressions of Nrf2, HO‐1, and NOQ1, while having no impact on Keap1 expression. However, Nrf2 silencing significantly abrogated BBR's ameliorative effect on cell apoptosis, inflammatory cytokine production, and the pyroptosis markers NLRP3, ASC, and GSDMD‐N. Through molecular docking, Su et al. discovered that BBR possesses the capability to target Nrf2, thereby modulating metabolism and exhibiting a range of beneficial effects, including hypoglycemic, lipid‐regulating, anti‐inflammatory, antioxidant, and immune regulatory properties.^40^ Li et al. demonstrated that BBR directly bind with Nrf2, inhibit the interaction between Keap1 and Nrf2, ands further suppress ferroptosis in Alzheimer's disease.^18^ To further investigate whether BBR directly interacts with Nrf2 signaling, molecular docking analysis in our current study indicated a direct binding between BBR and Keap1, suggesting that the binding energy of BBR to the key nodes of the Nrf2 signaling pathway varies in different disease models. Subsequently, Co‐IP assay was employed to definitively demonstrate that BBR could disrupt the interaction between Nrf2 and Keap1. qRT‐PCR examination elucidated that BBR significantly increased the mRNA levels of HO‐1 and NOQ1 but did not significantly alter Nrf2 and Keap1 mRNA levels in LPS treated GES‐1 cells, suggesting that BBR might affect Nrf2 degradation. CHX is a small molecule derived from *Streptomyces griseus* that acts as fungicide and is known to suppress protein degradation. MG132 has been used as a proteasome inhibitor on cells. Using CHX or MG132, the protein synthesis or degradation inhibition in GES‐1 cells was achieved, and the effect of BBR on Nrf2 protein stability was further determined. Together with the ubiquitination data, we confirmed that BBR inhibited Nrf2 ubiquitination and degradation. Furthermore, we observed that BBR promotes Nrf2 nuclear translocation, evidenced by increased nuclear Nrf2 protein levels in GES‐1 cells. These data indicate that BBR binds to Keap1, inhibiting Nrf2 ubiquitination and activating the Keap1/Nrf2 signaling pathway. Finally, intraperitoneal injection of the Nrf2 inhibitor ML385 reversed the protective effects of BBR on histopathological damage, inflammation, and pyroptosis, and abrogated the activation of Nrf2 signaling induced by BBR. Collectively, our data suggest that BBR inhibits pyroptosis and inflammation in sepsis‐induced acute gastric injury through the activation of the Keap1/Nrf2 signaling pathway.

In summary, our research offers compelling evidence that berberine (BBR) exerts profound protective effects against sepsis‐induced acute gastric injury in mice and LPS‐stimulated GES‐1 cells. Specifically, BBR binds to Keap1, inhibiting the ubiquitination of Nrf2, thereby activating the Keap1/Nrf2 signaling pathway. This activation ultimately contributes to BBR's protective role. Our findings suggest that BBR exhibits promising potential as a preventive agent against acute gastric injury, positioning it as a viable candidate for the development of innovative therapeutic strategies.

## CONFLICT OF INTEREST STATEMENT

The authors declare no conflicts of interest.

## Supporting information


**FIGURE S1:** Assessment of the effects of BBR on normal mice. (A) The quantitative analysis of serum ALT and AST levels in mice treated with 100 mg/kg BBR and untreated control mice. (B) H&E‐stained sections from the heart, liver, lung, kidney, and gastric tissue of mice treated with 100 mg/kg BBR and untreated control mice (200×). Scale bar: 100 μm. *N* = 6.
**FIGURE S2:** Gastric damage and inflammation at different time points in sepsis‐related mouse acute gastric injury. (A) H&E staining on gastric tissue sections after modeling for 6, 12, and 24 h (200×). Scale bar: 100 μm. (B) ELISA detection of TNF‐α, IL‐18, and IL‐1β in gastric tissues after modeling for 6, 12, and 24 h. *N* = 6. **p < 0.01 versus control group.
