# Supplementary material for: Berberine safeguards sepsis‐triggered acute gastric damage and inhibits pyroptosis in gastric epithelial cells via suppressing the ubiquitination and degradation of Nrf2
Source: Kaohsiung J Med Sci. 2024 Nov 1;40(11):1006–19. doi: 10.1002/kjm2.12900 (PMC11895072; doi:10.1002/kjm2.12900)
Supplement: Supplementary file 1 — FIGURE S1: Assessment of the effects of BBR on normal mice. (A) The quantitative analysis of serum ALT and AST levels in mice treated with 100 mg/kg BBR and untreated control mice. (B) H&E‐stained sections from the heart, liver, lung, kidney, and gastric tissue of mice treated with 100 mg/kg BBR and untreated control mice (200×). Scale bar: 100 μm. N = 6. FIGURE S2: Gastric damage and inflammation at different time points in sepsis‐related mouse acute gastric injury. (A) H&E staining on gastric tissue sections after modeling for 6, 12, and 24 h (200×). Scale bar: 100 μm. (B) ELISA detection of TNF‐α, IL‐18, and IL‐1β in gastric tissues after modeling for 6, 12, and 24 h. N = 6. **p < 0.01 versus control group. [file KJM2-40-1006-s001.docx]

**
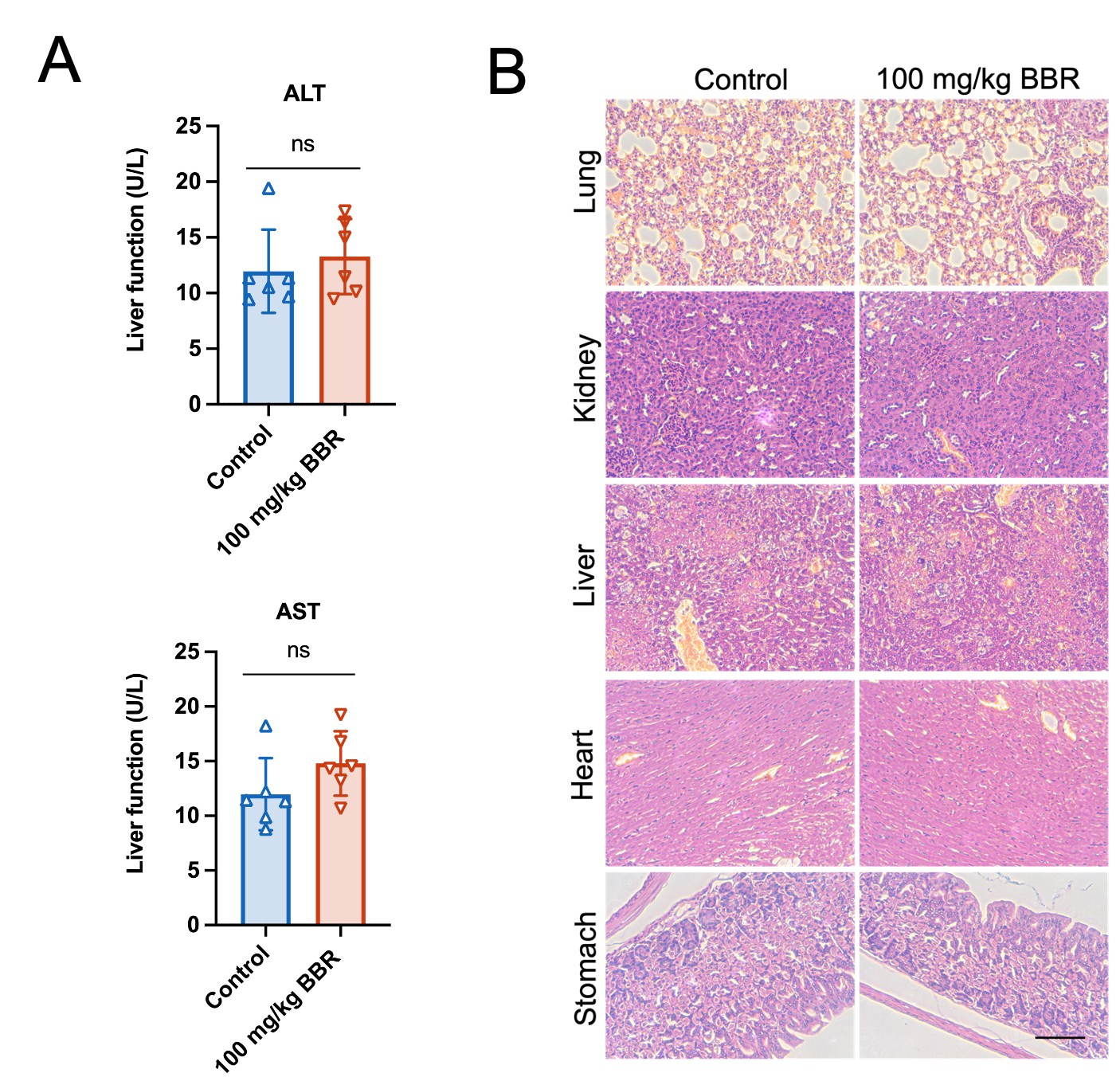
**

**Supplementary Figure 1. Assessment of the effects of BBR on normal mice.** (A) The quantitative analysis of serum ALT and AST levels in mice treated with 100 mg/kg BBR and untreated control mice. (B) H&E-stained sections from the heart, liver, lung, kidney, and gastric tissue of mice treated with 100 mg/kg BBR and untreated control mice (200×). Scar bar: 100 μm. N = 6.

**
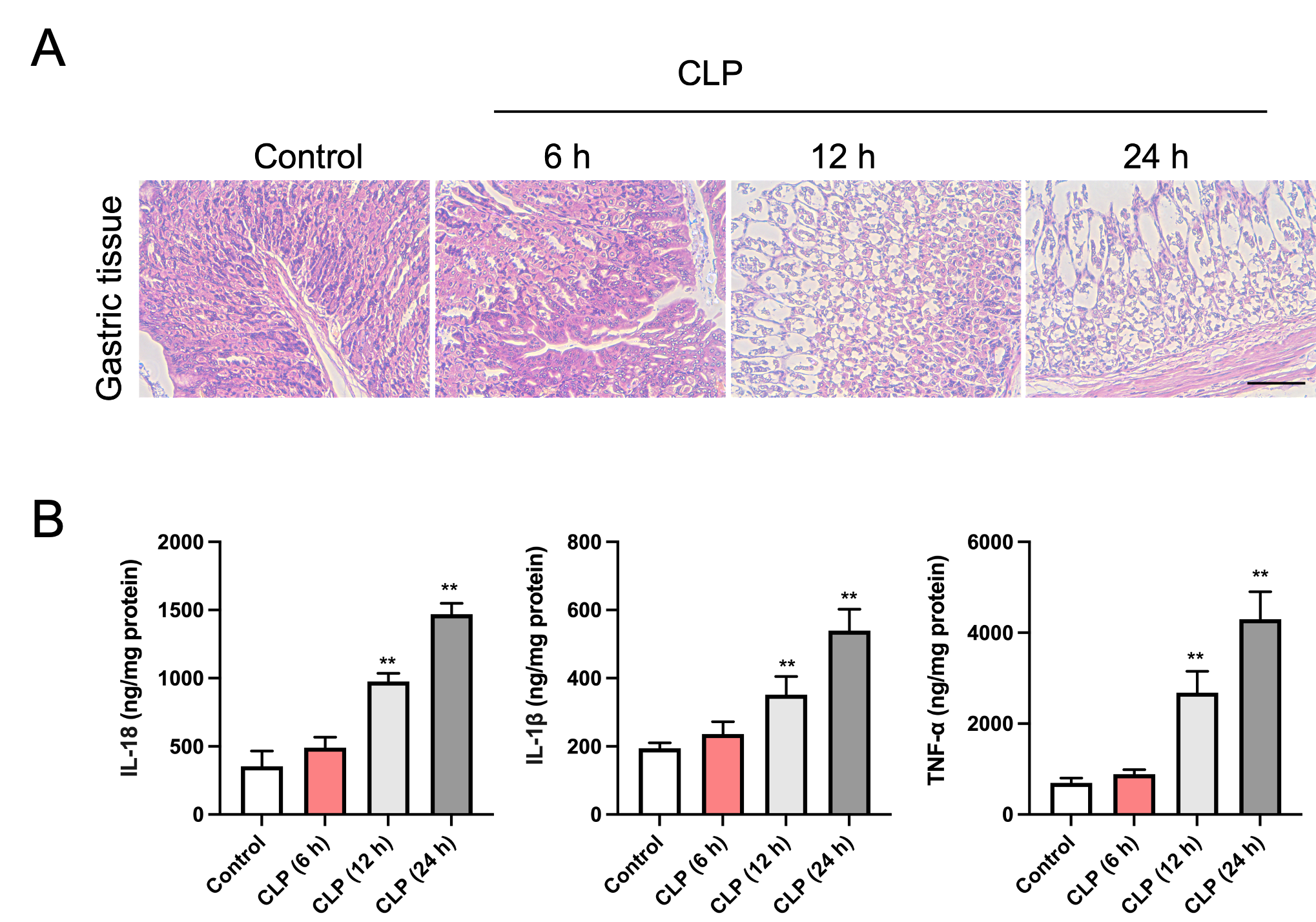
**

**Supplementary Figure 2. Gastric damage and inflammation at different time points in sepsis-related mouse acute gastric injury.** (A) H&E staining on gastric tissue sections after modeling for 6, 12, and 24 h (200×). Scar bar: 100 μm. (B) ELISA detection of TNF-α, IL-18 and IL-1β in gastric tissues after modeling for 6, 12, and 24 h. N = 6. ^**^*P*<0.01 *vs*. control group.
